# Soft Sweeps III: The Signature of Positive Selection from Recurrent Mutation

**DOI:** 10.1371/journal.pgen.0020186

**Published:** 2006-12-15

**Authors:** Pleuni S Pennings, Joachim Hermisson

**Affiliations:** Section of Evolutionary Biology, Department Biology II, Ludwig-Maximilians-University Munich, Planegg-Martinsried, Germany; University of Chicago, United States of America

## Abstract

Polymorphism data can be used to identify loci at which a beneficial allele has recently gone to fixation, given that an accurate description of the signature of selection is available. In the classical model that is used, a favored allele derives from a single mutational origin. This ignores the fact that beneficial alleles can enter a population recurrently by mutation during the selective phase. In this study, we present a combination of analytical and simulation results to demonstrate the effect of adaptation from recurrent mutation on summary statistics for polymorphism data from a linked neutral locus. We also analyze the power of standard neutrality tests based on the frequency spectrum or on linkage disequilibrium (LD) under this scenario. For recurrent beneficial mutation at biologically realistic rates, we find substantial deviations from the classical pattern of a selective sweep from a single new mutation. Deviations from neutrality in the level of polymorphism and in the frequency spectrum are much less pronounced than in the classical sweep pattern. In contrast, for levels of LD, the signature is even stronger if recurrent beneficial mutation plays a role. We suggest a variant of existing LD tests that increases their power to detect this signature.

## Synopsis

Populations adapt to their environment through fixation of beneficial alleles. Such fixation events leave a signature in neutral DNA variation of the population. An accurate description of this signature, also called a selective sweep, can be used to identify genes that have been involved in recent adaptations. The classical model of a selective sweep assumes that the beneficial allele was created only once by mutation, whereas the authors have shown, in a previous paper, that this assumption does not always hold. If a substitution involves multiple copies of an allele that have originated by independent mutation, it leads to a different signature, which the authors call a soft selective sweep. In this study, Pennings and Hermisson use analytical tools and coalescent simulations to describe this soft-sweep pattern. They show that this pattern is characterized by strong linkage disequilibrium. They also analyze the power of standard tests of neutrality to detect this pattern and suggest a variant of existing linkage-disequilibrium–based tests that increase the power to detect positive selection in the form of a soft selective sweep.

## Introduction

Patterns of DNA polymorphism can be used to infer the processes that have played a role in the evolutionary history of a population. A process that is of primary interest to evolutionary biologists is directional selection, and the pattern that is left by it, a so-called selective sweep, has received a lot of attention in the literature since it was first described by Maynard-Smith and Haigh [1]. By now, this pattern is well studied, at least for a simplified model, which assumes that a single adaptive mutation increases in frequency under constant selection pressure in a panmictic population of constant size (e.g., [2–7]). The signature that is created if these assumptions are met is characterized by 1) low polymorphism around the selected site, 2) an excess of low-frequency variants both at the locus itself and in the flanking regions, 3) an excess of high-frequency variants only in the flanking regions, and 4) strong linkage disequilibrium (LD) in the flanking regions, but no LD between mutations on opposite sides of the selected locus. There is a body of statistical tests based on these characteristics (e.g., [8–12]), which have been used in a large number of studies seeking to identify loci that have undergone directional selection (e.g., [13–19]).

One assumption of the simplified model is that only descendants of a single copy of the beneficial allele contribute to fixation. This may be different if 1) selection acts on the standing genetic variation or 2) adaptation occurs from recurrent mutation or migration. If (descendents of) multiple copies of a beneficial allele are involved in its fixation, this has consequences for the signature of selection. We therefore call such a signature a “soft selective sweep” and distinguish it from the classical pattern of a “hard sweep,” in which only a single copy is involved [20].

Adaptation from the standing genetic variation has been described in a series of recent articles [20–23]. Substantial changes to the classical hard sweep are observed, in particular, if the allele had been neutral prior to the onset of positive selection. The second scenario, adaptation from recurrent mutation or migration, was analyzed in [24]. It turns out that soft selective sweeps from recurrent mutation are relevant if Θ*_b_* > 0.01 (where Θ*_b_* = 2*N_e_u_b_* is the population mutation parameter for the beneficial allele). Soft sweeps, under these conditions, are therefore likely if either the (inbreeding) effective population size *N_e_* is large or if the allelic mutation rate *u_b_* is high. For example, Li and Stephan [25], estimate that for African *Drosophila melanogaster,* which has high *N_e_,* the mutation parameter per site is about 0.05. Since the allelic mutation rate Θ*_b_* will usually be equal to or higher than the rate per site, soft sweeps from recurrent mutation should be frequent for this species. A large Θ*_b_* is also expected, even for populations with moderate or small *N_e_,* if adaptation involves a loss- or reduction-of-function mutation. Adaptive loss-of-function mutations have recently been identified in many species, such as humans (e.g., [13,26,27]), D. melanogaster [28], Arabidopsis thaliana [29], and rice [30].

In this study, we describe how a soft sweep from recurrent mutation affects a neutral locus at some recombinational distance from the selected locus and which tests can be employed to detect soft sweeps. We will see that the deviation from the classical hard-sweep pattern is even stronger than for adaptation from standing genetic variation. The reason is that haplotypes that are associated with different mutational origins of a beneficial allele are truly independent. In contrast, multiple copies of the beneficial allele that segregate in the standing genetic variation may still be identical by descent.

In the following, we first derive formulas for the site-frequency spectrum and the number of haplotypes at a locus tightly linked to the selected site. We compare the effects of recombination and recurrent beneficial mutation on the polymorphism pattern and explain the differences from the different timing of these events in the coalescent of a sample. In a second step, we describe the combined effect of recurrent mutation and recombination on summary statistics for DNA polymorphism at a linked neutral locus. Finally, we present a power analysis of various neutrality tests. Recent soft sweeps from recurrent mutation can be detected very well using LD-based tests, but not using frequency-spectrum–based tests. We show that older sweeps can also be revealed by LD tests if information from a recently derived sister population is available.

## Methods

We consider a haploid population of constant effective size *N_e_*. At a locus under selection, there are two alleles, an ancestral allele *b* with fitness 1 and a beneficial variant *B* with fitness 1 + *s*. The *B* allele may also correspond to a class of physiologically equivalent alleles, in which case we assume that these alleles are at the same locus and tightly linked. Mutation from *b* to *B* happens at rate *u_b_;* back mutation is ignored. We study the polymorphism pattern at a neutral locus at a recombinational distance *r* from the selected site. The neutral mutation rate at the study locus is *u_n_,* and we assume an infinite-sites model for this locus. Recombination within the neutral locus is denoted by *r_n_*. We define population level parameters as Θ*_b_* = 2*N_e_u_b_* (beneficial mutation rate for the allele), Θ*_n_* = 2*N_e_u_n_* (neutral mutation rate), *R* = *N_e_r* (recombination rate between the selected and neutral locus), *R_n_* = *N_e_r_n_* (recombination rate between the two ends of the neutral locus), and *α* = *N_e_s* (strength of selection). If we set *r* = *r_n_* = 0, and assume that Θ*_n_* is so high that two random haplotypes from the population are always different, the model is identical to the model from Pennings and Hermisson [24].

We use a coalescent framework and define *τ* as the time in the past before fixation of the *B* allele. The frequency of the *B* allele is denoted by *x_τ_*. The time from the first origin of a *B* allele that will contribute to fixation and *x_τ_* = 1 is referred to as the selective phase, and the length of the selective phase is *T_fix_* generations. In the selective phase, the population can be separated in a growing *B* part and a shrinking *b* part (forward in time). We can therefore use a structured coalescent to derive the sampling distributions at the neutral locus [2]. If a *b* → *B* mutation happens during the selective phase, a new lineage enters the *B* part of the population. If this happens in the history of a sample, we call it a soft sweep. In a coalescent view, lineages in a sample at the selected locus can coalesce with each other or they can escape the *B* population by mutation. At the neutral locus, a lineage can also escape by recombination (see Figure 1).

**Figure 1:**
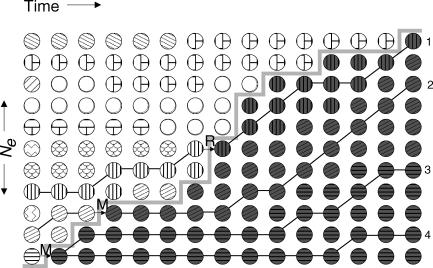
Selective Sweep with Recurrent Mutation and Recombination in a Schematic Wright-Fisher Model Circles represent individuals in the population; the different patterns indicate independent haplotypes at the neutral locus. An individual is dark grey when it is associated with the beneficial allele *B* at the selected site, and white when it is associated with the ancestral *b* allele. The *B* allele arises two times by independent mutations (indicated by *M*); individuals then change their color from white to grey, but keep their pattern. Similarly, a *b* lineage can recombine onto a *B* allele (indicated by R), in which case the individual also changes its color and keeps its pattern. Directly after fixation (*t* = 0), we take a sample of three individuals. If the sample would contain individuals (2, 3, 4), it would have two ancestral haplotypes because it is a soft sweep. If the sample would be (1, 3, 4) it would also contain two ancestral haplotypes, but this time because of recombination. In a coalescent view, both 1 and 2 escape the *B* part of the population.

### Simulations of Positive Selection

We used the program of Kim and Stephan [11] to which we added the possibility of recurrent beneficial mutation. In the simulations, a neutral fragment is affected by the fixation of a beneficial allele at a nearby selected site. The fragment starts directly next to the selected site (at distance *R* = 0), or at one of five recombinational distances away from it (*R* = 10; 20; 100; 200; 600). Recombination and neutral mutation within the neutral fragment happens at rate *R_n_* = 10 and Θ*_n_* = 10 (except for some additional simulations described in the text). This corresponds to a 500–base pair (bp)-long fragment if the per nucleotide mutation rate and recombination rate are both 1 × 10^−8^, and the population size is *N_e_* = 1,000,000. For all our figures, we assumed strong selection (*α* = 10,000). Results from additional simulation runs with *α* = 1,000 are described in the text. For all figures, we ran 10,000 simulations per parameter combination.

A sample taken at *tN_e_* generations after fixation of the beneficial *B* allele is simulated. For this, a coalescent graph with recombination is built backwards in time in three phases. The simulation starts with a standard ancestral recombination graph during the neutral phase from *tN_e_* generations after fixation to fixation, followed by a structured coalescent during the selective phase, and finally a second neutral phase with an ancestral recombination graph before the origin of the *B* allele. This last phase lasts until all lineages have coalesced.

Backward in time, lineages can coalesce, they can recombine, and they can mutate from *B* to *b*. During the neutral phases, coalescence can happen between all lineages and only recombination within the fragment is modeled. During the selective phase, coalescence can only happen between lineages in the same part (*b* or *B*) of the population. Recombination can happen either within the fragment or between the fragment and the selected site. In the latter case, it is only of interest whether the lineage changes the subpopulation that it belongs to; lineages can recombine from the *B* subpopulation into the *b* subpopulation, and vice versa. When the breakpoint of the recombination event is within the fragment, the lineage splits in two, and the part that is farthest from the selected site may change the subpopulation that it is in. Mutation from *B* to *b* (in the backward direction) can only happen during the selective phase, with the probability given in Equation 4. Mutation from *b* to *B* is ignored.

The structured coalescent during the selective phase is conditioned on the frequency of the beneficial allele *x*_τ_(0 < τ < *T_fix_*), which is obtained by conducting for each replicate an independent forward-in-time simulation using a Wright-Fisher model with recurrent beneficial mutation. In the model without recurrent mutation (hard-sweep model), we inserted a single beneficial mutant in the population. Conditioning on fixation was done by discarding all runs in which the *B* allele did not go to fixation. Tajima's *D* is only defined if there is at least one polymorphic site, and Kelly's *ZnS* is only defined if there are at least two polymorphic sites. For the means and standard deviations that are shown in Results, runs for which a statistic is not defined were taken out. The code was checked by comparing the probability of a soft sweep in backward-in-time simulations against results from forward-in-time simulations.

### Power Analysis

Outcomes of the simulations with positive selection were compared with the critical values from neutral simulations with the same number of polymorphic sites *(S),* to check for significant deviations from the neutral expectation. Critical values were obtained from Hudson's ms program [31], conditional on the number of polymorphic sites (as in, e.g., [32,33]). Because we expect deviations of Tajima's *D* test in two directions, we used the test as a two-sided test, unlike Przeworski [33], but like Depaulis et al. [34]. Using the neutral simulations, we determined the *D* value at 2.5% and 97.5% of the distribution for each value of *S*. For the other tests (Fay and Wu's *H,* haplotype *K,* and Kelly's *ZnS* test), we expect deviations due to positive selection in only one direction. They were therefore implemented as one-sided tests. We assumed no recombination in the neutral simulations, which is the conservative choice because it will lead to stronger LD. For the power analysis, we do not exclude runs in which there are no polymorphic sites, unlike Przeworski [33], but like Depaulis et al. [34]. This is because we are interested in the probability that we can detect an episode of selection with a given neutrality test. If there are no polymorphic sites (*S* = 0), selection cannot be detected with a test that is conditioned on *S*.

For the tests for which we excluded new mutations, we used Kim and Stephan's program [11], conditional on *S,* to obtain the critical values for each *t* value (time after fixation). In these simulations, no mutations were allowed on in the last *tN_e_* generations of the coalescent tree, and we again assumed no recombination.

## Results

### Polymorphism Pattern at a Tightly Linked Locus

Approximate analytical results are possible for the expected polymorphism pattern at a locus that is tightly linked to the selected site (*r* = *r_n_* = 0). In Pennings and Hermisson [24], we were interested in the number of ancestors a sample has at the beginning of the selective phase (forward in time). Each ancestor corresponds to an independent ancestral haplotype, i.e., an independent random pick from the ancestral population, before the onset of positive selection. Note that these draws do not necessarily result in different haplotypes. We showed that the distribution of independent ancestral haplotypes in a sample that is taken after fixation of the beneficial allele is approximately given by the Ewens sampling formula. If we want to determine the frequency spectrum of polymorphic sites, we need to trace the history of the sample farther back in time.

In the following, we assume that the population has been in neutral equilibrium prior to the single episode of positive selection that we consider (see Analytical Derivations for some added generality on this point). Because the relationship between the ancestral haplotypes is then given by a neutral coalescent, we need to combine the Ewens sampling formula for the distribution of ancestral haplotypes with a neutral coalescent for the history of these ancestral haplotypes. We find that the probability that a mutation is carried by ℓ individuals out of *n* is

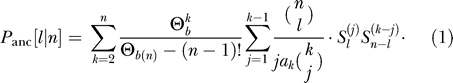
(with 
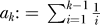
 ; 
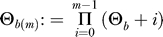
 ; and 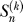 is the nonnegative Stirling number of first kind). The derivation is in Analytical Derivations. In Figure 2, we compare this prediction with simulation results. For the approximation, we have ignored neutral mutations during the selective phase, but they are included in the simulations. As can be seen in Figure 2, the approximation holds very well for large *α*. For smaller *α,* an excess of singletons becomes visible as neutral mutations accumulate during the selective phase (unpublished data).

**Figure 2:**
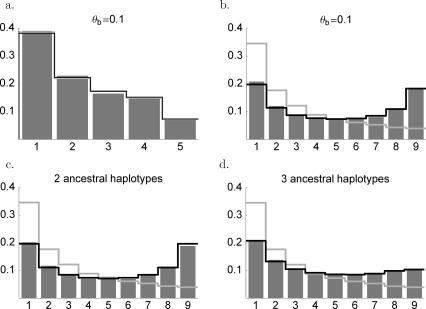
Frequency Spectrum at Fixation Simulations are done without recombination, but with new mutations during the selective phase. The bars are simulation results; the black lines are the predictions from Equation 1. The light grey line is the frequency spectrum under neutrality. (A) Frequency spectrum at the time of fixation in a sample of 10, θ*_b_* = 0.1. If there is only one ancestral haplotype (hard sweep), there will be no polymorphic sites, so conditioning on soft sweeps does not change the frequency spectrum. (B) Same as (A), but now polarized (see text). (C) Same as (B), but after a soft sweep with exactly two ancestral haplotypes. (This frequency spectrum is symmetrical.) (D) Same as (B), but after a soft sweep with three ancestral haplotypes.

A few things become clear from Figure 2. First, Figure 2A shows that the folded frequency spectrum after a sweep for Θ*_b_* = 0.1 is virtually the same as the neutral expectation. In fact, it is exactly the same if there are exactly two ancestral haplotypes (which is the most common outcome for Θ*_b_* = 0.1). Second, the polarized (or unfolded) frequency spectrum is very different from the neutral expectation (see Figure 2B). There is a clear excess of high-frequency variants when there are two or three ancestral haplotypes in the sample (Figure 2C and 2D).

If there are two ancestral haplotypes, the polarized frequency spectrum is symmetrical. In this case, sites can only stay polymorphic if one variant is associated with the first beneficial mutation and the other is associated with the other beneficial mutation. The beneficial mutations, and therefore the neutral variants, must have complementary frequencies. They therefore have equal probability to end up in the major or in the minor haplotype, which results in the observed symmetry.

The number of distinct haplotypes can be lower than the number of independent ancestral haplotypes as defined in Pennings and Hermisson [24], because there is a chance that independent ancestral haplotypes are identical. Whether ancestral haplotypes are the same or different is an infinite alleles problem and can therefore be described by a coalescent with killings [35]. The number of distinct haplotypes, if the number of ancestral haplotypes is known, is given by the Ewens sampling formula. To know the number of distinct haplotypes in a sample, we therefore need to combine two Ewens sampling formulas, one that tells us the number of ancestral haplotypes and one that tells us how many of these are distinct. The probability that there are ℓ distinct haplotypes in a sample is given by

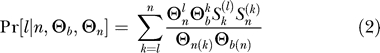
(the derivation is given in Analytical Derivations). In Figure 3, the prediction from Equation 2 is compared with simulation results. For Θ*_n_* → ∞, the probability that two ancestral haplotypes are different is 1, and the number of distinct haplotypes is the same as the number of ancestral haplotypes. For lower values of Θ*_n_,* there may be fewer distinct than ancestral haplotypes. The difference is clearest for the categories with many haplotypes, because if many haplotypes are sampled from the population, it becomes less likely that they are all different. If there are only two ancestral haplotypes, they are distinct with probability 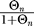 (which is ≈ 0.91 for Θ*_n_* = 10). The expected number of haplotypes under neutrality, for Θ*_n_* = 10, is about 11 haplotypes. The number of distinct haplotypes in the sample after a soft sweep is therefore still much lower than the neutral expectation.

**Figure 3:**
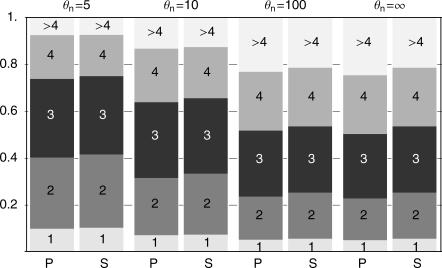
Probability of Finding 1, 2, 3, etc., Distinct Haplotypes Depending on the Neutral Mutation Rate θ*_n_ ,* in a Sample of 20 at the Time of Fixation, with θ*_b_* = 1.0 Predictions from Equation 2 are labeled P; simulation results are labeled S. Simulations are done without recombination and neutral mutations during the selective phase.

### The Footprint of Selection at a Linked Locus

To describe the footprint of selection at a neutral locus at some distance from the selected locus, we need also to take recombination into account. When we trace the ancestry of a sample back in time, three things of interest can happen: 1) Two lineages can coalesce when they find a common ancestor, 2) one lineage can choose as its ancestor a *b* individual that has mutated into a *B* individual and thus escape the sweep (note that mutation happens at the associated selected locus and not at the neutral locus that we follow), or 3) one lineage can recombine onto a *b* background. We assume the population is large and can therefore set *x_τ_*_−1_ ≈ *x_τ_*. The probabilities of coalescence, mutation, and recombination in a generation *τ,* when there are *k* lineages left, are given by:

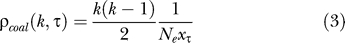


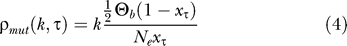


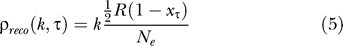
(e.g., [24,36]). Consider now a sample of size two. We are interested in the timing of the first event in the coalescence process of this sample and in the type of this event. The probability that the first event occurred *τ* generations ago and that this event was a beneficial mutation is

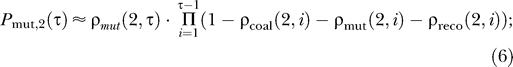
where the product is the probability that no event has happened until *τ* − 1. Equivalent equations hold for coalescence and recombination.

Figure 4 shows how the probabilities for each of these events and the frequency of the *B* allele change in time. It shows clearly that mutation events happen early in the selective phase, just like coalescence events. Recombination, on the other hand, happens later. We can see from Equations 1–3 what causes this difference. Both the coalescence probability and the mutation probability have a 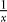 term, but the recombination probability does not. This 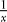 term causes the coalescence and mutation probabilities to rise steeply when the frequency of the *B* alleles goes down. The recombination probability has only a (1 − *x*) term, which means it will go up when *x* goes down, but much less so.

**Figure 4:**
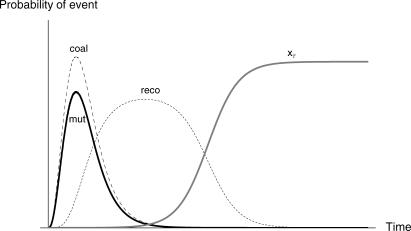
Timing of Coalescence, Recombination, and Mutation Events during the Selective Phase in a Sample of Two This plot shows the probability that recombination (reco), mutation (mut), or coalescence (coal) happens during the selective phase when we trace the ancestry of a sample of size 2 back in time. The parameter values for this plot are chosen so that the timing of the three events is made clear; no importance should be given to the relative heights of the curves. The curve with label *x_τ_* shows the frequency of the *B* allele in the population.

Backward in time, most recombination events happen at a time in which it is unlikely that coalescence events have happened already. This separation in time of recombination and coalescence is used already in Maynard-Smith and Haigh [1]. Durett and Schweinsberg [5] and Etheridge et al. [6] show that this is valid as a first-order approximation in *α*. Recombination therefore tends to make single lineages escape and produces strongly unbalanced trees and polymorphism patterns with an excess of low-frequency alleles. In contrast, the distributions for mutation and coalescence events fully overlap, which means that for larger samples, it is likely that some coalescence events have happened before a mutation event and some after. As a consequence of this timing, family sizes of an escaping lineage can be anything from just one to almost all lineages. Mutation will therefore create a very different frequency spectrum (as seen in Figure 2) than recombination.

That a recurrent beneficial mutation tends to happen early in the selective phase can also be understood in a forward-in-time picture. First, to appear in a sample, the mutation needs to reach a high frequency and this is more likely if it happens quickly after the first mutation. Second, early mutants have a higher probability to escape stochastic loss because the mean fitness in the population is still lower and therefore the relative fitness of a mutant is higher. Third, simply more *b* → *B* mutations happen in the beginning of the selective phase because there are more *b* alleles in the population at this time.

### The Effect of Recurrent Mutation on Summary Statistics

To describe the effect of positive selection under recurrent mutation on the polymorphism pattern, we consider a sample from a linked neutral locus that is taken at fixation of the beneficial allele. We derive analytical approximations for the number of pairwise differences *π* and the number of polymorphic sites *S*. These approximations are complemented by coalescent simulations for *π, S,* Tajima's *D,* Kelly's *ZnS,* and the number of haplotypes *K* under neutrality, and three scenarios for a selective sweep (Figure 5): 1) a standard sweep model without recurrent mutation (hard sweep), 2) a sweep model with Θ*_b_* = 0.1 where we conditioned on soft sweeps (i.e., only those simulation runs were considered in which a soft sweep had happened), and 3) a sweep model with Θ*_b_* = 1.0. About 95% of all sweeps are soft in this case.

**Figure 5:**
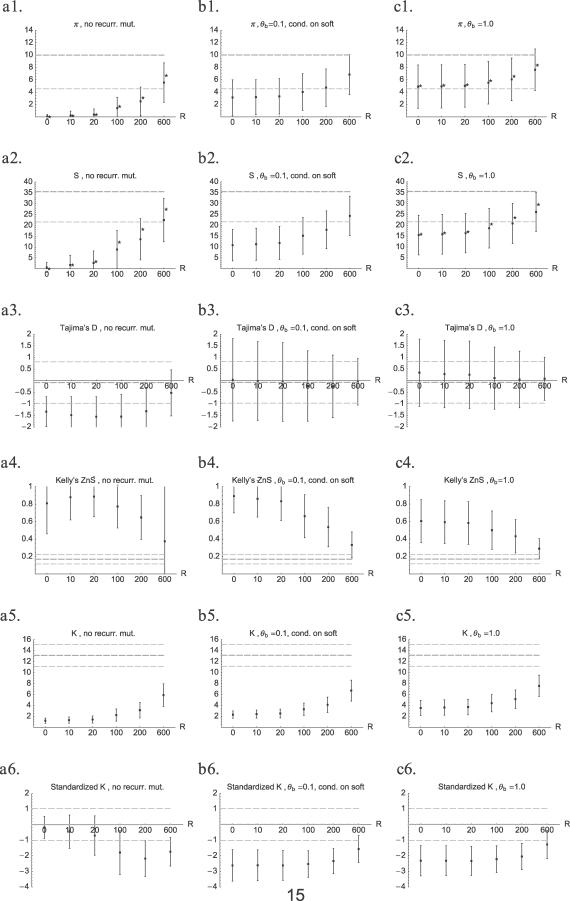
Means (± One Standard Deviation) of Summary Statistics in a Sample Taken at Fixation of a Beneficial Allele The *x-*axis shows the distance from the selected site in units of *R* = *N_e_r*. The left column (A1–A6) shows hard sweeps (no recurrent mutation [no recurr. mut.]); the middle column (B1–B6) shows only soft sweeps (cond. on soft) for beneficial mutation rate θ*_b_* = 0.1; and the right column (C1–C6) shows averages over all sweeps (hard or soft) for θ*_b_* = 1.0. The statistics are from top to bottom are: 1) mean number of pairwise differences (π), 2) number of polymorphic sites *(S),* 3) Tajima's *D,* 4) Kelly's *ZnS,* 5) number of haplotypes *K,* and 6) standardized *K* (see text). The grey lines indicate means (thick dashed line) ± one standard deviation (thin dashed line) under neutrality. In the plots for *π* and *S,* asterisks (*) depict predicted values based on Equations 8 and 18. Parameters are as described in Methods.

For our analytical approximations, we ignore neutral mutation during the selective phase. Following our results from the last section, we also assume a complete separation in time between recombination on the one hand and coalescence and beneficial mutation on the other hand. This means that, in a coalescent framework, recombination during the selective phase is considered first, while coalescence and beneficial mutation events all occur right at the start of this phase. Finally, we ignore all events (recombination or coalescence) in the *b* part of the population. Coalescent simulations treat the full model, without any of these approximations.

#### Pairwise difference (*π*)*.*

Under the above assumptions, a pairwise difference can only occur if one of the two lineages escapes the *B* part of the population by recombination or mutation. If this happens, the probability that the site is polymorphic is the same as it was under neutrality. Recombination can happen anywhere during the selective phase, with rate 2*r* (for two lineages) per generation. We are, however, only interested in recombination events that involve *b* alleles, which will be the case for half of the events. Namely, averaged over the time of the selective phase, the fraction of *b* alleles in the population is 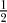 . The number of relevant recombination events is therefore Poisson distributed with parameter 2*rT_fix_*/2, where *T_fix_* is the fixation time. The probability that at least one recombination event happens is therefore

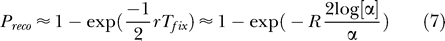
where we use 
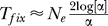
 [20]. This result coincides with Etheridge et al. [6] and Nielsen et al. [12]. If no recombination with a *b* lineage has happened, there is a probability 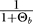 that the lineages coalesce before a beneficial mutation happens [24]. The probability that neither recombination nor mutation happens is then, 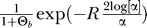 , and the expected *π* given the neutral *π_n_* is

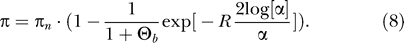
In Figure 5, we compare this result with simulation data. The approximation works well as long as *R* is not too large (Figure 5A1 and 5C1). For large *R,* lineages that have escaped from the *B* part of the population through recombination may enter it again through another recombination event. This is ignored in the analytical approximation, which therefore overestimates *π* at large distances. The effect of recurrent mutation on the signature in *π* is straightforward: since for a soft sweep polymorphism is even maintained at *R* = 0, the depth of the reduction in *π* is reduced (Figure 5B1 and 5C1).

#### The number of polymorphic sites (*S*).

In our approximation, the number of polymorphic sites depends only on the number *m* of lineages at the start of the selective phase. These ancestral lineages are related by a neutral coalescent, and for *m* ancestors, the expected number of polymorphic sites is Θ*_n_a_m_*. For *m,* we need to add up all lineages that escape the sweep by either recombination or beneficial mutation. The derivation and the result are given in Analytical Derivations. The prediction is compared with simulation data in Figure 5A2 and 5C2. For *R* > 0, the approximation is a bit worse than for *π*. The reason is that the separation in time of recombination and coalescence is less good for larger samples.

Just as for *π,* the footprint in *S* is weakened due to soft sweeps. When scanning for sweeps, low *S* or *π* is often the first indication that there may have been a sweep near the studied fragment. It is therefore important to realize that, contrary to a hard sweep, a soft sweep will usually not be characterized by a very low *π* or *S*.

#### Tajima's *D*.

Tajima's *D* is a frequency-spectrum–based test statistic [8]. Roughly, it measures the contribution of intermediate frequency mutations to the total number of mutations. When this contribution is higher than expected, Tajima's *D* is positive, when lower, *D* is negative. After a hard sweep, Tajima's *D* tends to be very negative in the flanking regions, because recombination produces an excess of low-frequency mutations. In contrast, this effect is almost not visible after a soft sweep. In fact, for soft sweeps, the mean *D* is not much different from 0. However, the standard deviation of *D* is greatly increased as compared with neutrality or the standard hard sweep. Both these phenomena can easily be understood. As we have already seen in our calculations for *R* = 0 above, the (average) folded frequency spectrum after a soft sweep is very similar to the neutral spectrum. As also predicted there, the average *D* close to the selected site is even positive for large Θ*_b_* (Figure 5C3). The large variance is a consequence of the timing of beneficial mutation events as shown in Figure 4. Since mutation and coalescence can occur in any order, there is a wide range of possible family sizes that can escape the sweep through mutation, which can result in either a very negative *D* (if a single lineage escapes) or a positive *D* (if a larger family escapes). As for a hard sweep, recombination reduces *D* in the flanking regions of a soft sweep. However, in the presence of polymorphism due to lineages that escape because of recurrent mutation, this effect is much reduced.

#### Kelly's *ZnS*.

Both soft and hard sweeps affect the shape of the coalescent tree of a sample and thereby the associations (LD) between neutral mutations that fall on that tree. One way to measure LD is by using Kelly's *ZnS* statistic [37], which is based on pairwise LD. Mutations that happen on the same branch in a tree cause high *ZnS* values. The range of values that *ZnS* can take is from 0 to 1, with higher values denoting stronger LD. From the plots (Figure 5A4-5C4), it looks as if both soft and hard sweeps show about the same result: *ZnS* is much higher than the neutral expectation. However, in this case, the plots show only part of the story. *ZnS* is only defined if there are two or more polymorphic sites. In the case of a hard sweep, many runs (about 90% directly next to the selected site) produced fewer than two polymorphic sites. For those runs, we could therefore not calculate *ZnS*. The runs with more than one polymorphism were mostly those where a recombination event had taken place, and this leads to high *ZnS* values. In the soft-sweep simulations, there were only few runs with fewer than two polymorphic sites (8% of the runs next to the selected site). After a soft sweep, *ZnS* is therefore also high if no recombination has taken place yet.

#### The number of haplotypes (*K*).

The number of haplotypes in a sample *K,* shown in Figure 5A5-5C5, is simply a count of the number of different sequences that are found in a sample [9]. Note that the number of haplotypes here is higher than in Figure 3, because of recombination (both between the selected and the neutral locus and within the neutral locus) and new neutral mutations. *K* is much lower than the neutral expectation everywhere for both hard and soft sweeps. However, close to the selected site for the hard sweep, this is mainly due to a low number of polymorphic sites *S* and not because of a strong haplotype structure. For example, if *S* = 1, there can be only two haplotypes; for *S* = 2, there can be either two or three haplotypes. In these cases, *K* is not a very informative statistic, at least if we already have the information about *S*. Away from the selected site, and everywhere for the higher Θ*_b_* values, *K* is low because of haplotype structure. To capture this effect, we have made an attempt to standardize the *K* values. Using neutral simulations, we have estimated the expectation and standard deviation of *K,* given a fixed number of polymorphic sites. We have defined K′ (standardized K) as K′ = 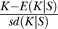 , and we define K′ = 0 if S < 2. The last row of panels in Figure 5 shows that K′ is lower than expected if K is low despite relatively high *S*. On the other hand, *K′* is not different from the neutral expectation if there are very few polymorphic sites.

### Power Analysis

Again using simulations, we have done a power analysis of two frequency-spectrum–based tests (Tajima's *D* and Fay and Wu's *H*) and two LD-based tests (number of haplotypes *K* and Kelly's *ZnS*). For a given set of parameters, the probability is estimated that a simulation run results in a significantly positive or negative test statistic. Critical values for the tests are obtained using simulations of a neutral model without recombination (for details, see Methods).

We did simulations for six scenarios: without recurrent mutation, Θ*_b_* values 0.1, 0.4, 1.0, and 4.0, and Θ*_b_* = 0.1 conditioned on a soft sweep. We have looked at these scenarios at seven different times after fixation of the beneficial allele: *t* = 0, 0.01, 0.05, 0.1, 0.2, 0.5, and 1.0 (time is measured in *N_e_* generations). We again looked at fragments at six recombinational distances from the selected site. The results are shown in Figures 6 and 7.

**Figure 6:**
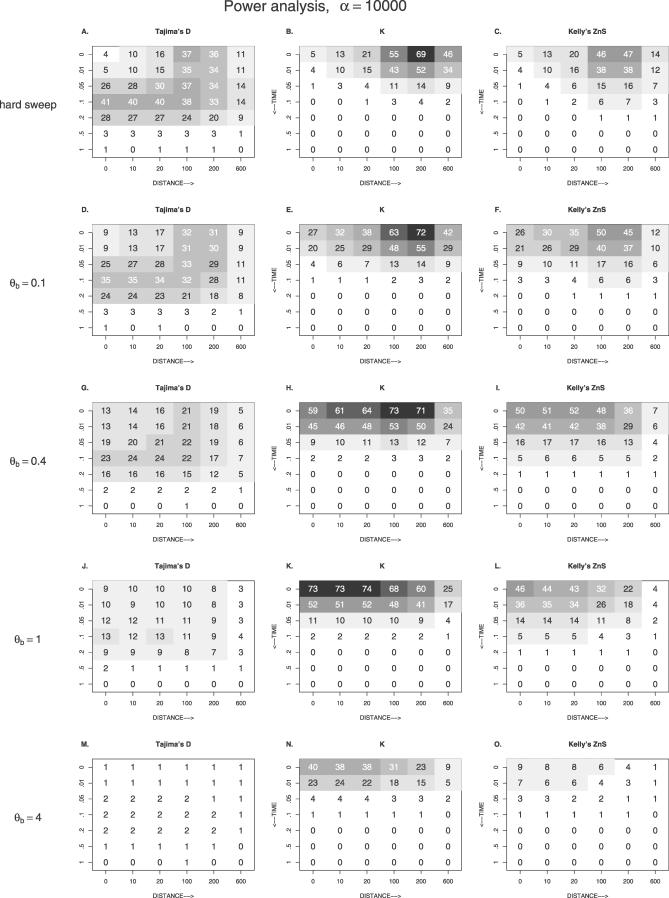
The Percentage of Simulation Runs That Yielded a Significant Test Statistic Depending on the Value of θ*_b_,* Other Parameters as Standard The *x-*axis shows the distance from the selected site in units of *R* = *N_e_r*. The *y-*axis shows the time since fixation of the *B* allele in units of *N_e_* generations.

**Figure 7:**
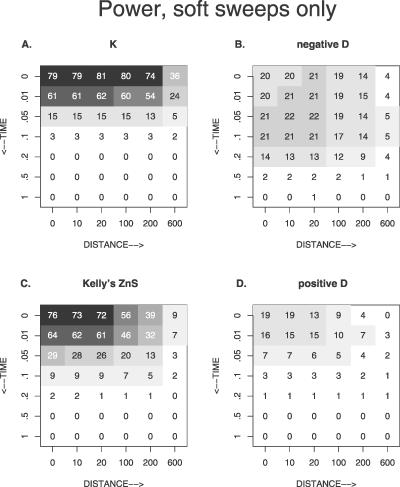
The Percentage of Simulation Runs That Yielded a Significant Test Statistic If We Condition on a Soft Sweep θ*_b_* = 0.1, other parameters as standard. The *x*-axis shows the distance from the selected site in units of *R* = *N_e_r*. The *y-*axis shows the time since fixation of the *B* allele in units of *Ne* generations.

#### Frequency-spectrum–based tests.

We conducted Tajima's *D* as a two-sided test and Fay and Wu's *H* [10] as a one-sided test. Results from Tajima's test are shown in Figures 6 and 7; results for Fay and Wu's *H* are unpublished data, and are only described below. For a classical hard sweep without recurrent mutation, frequency-spectrum–based tests have no power at the selected site, directly at fixation, simply because of lack of polymorphism. Tajima's *D* has moderately high power (up to 41%) to detect hard sweeps either at some distance (*R* = 100 or 200) from the selected site because of recombination, or at some time after fixation (*t* between 0.05 and 0.2), because of new mutations that have a low frequency. In Figure 6A, the region of high power shows as a dark quarter ring. When we increase the beneficial mutation rate, and thereby allow for soft sweeps to happen, and also if we condition on soft sweeps (see Figure 7), the power of Tajima's *D* goes down in the regions where the power was high before. At the same time, close to the selected site, where the power was low in the hard-sweep case, the power goes up. Directly next to the selected locus, the power reaches 20% (see Figure 7C). This is not surprising, even though the frequency spectrum after a soft sweep is expected to be similar to that under neutrality (see Figure 2A). It is the large variance of *D* after a soft sweep (see Figure 5) that causes these significantly negative *D* values. Since the mean *D* is not much different from 0, the large variance also causes significantly positive *D* values (19%), as is shown in Figure 7D.

Fay and Wu's *H* is negative if there is an excess of high-frequency–derived mutations, which is expected in the flanking regions of a selected site after a hard sweep. Fay and Wu's *H* therefore has high power in the flanking regions (up to 63%). However, with time, the power reduces quickly, because new mutations that accumulate will have low frequencies, and the high-frequency variants may be lost by drift [33]. For higher Θ*_b_* values, the power of *H* goes down in the flanking regions, but just as for Tajima's *D,* the power goes up (up to 34%) close to the selected site. In fact, we expect significant *H* values there, because the frequency spectrum close to the selected locus shows an excess of high-frequency–derived variants (see Figure 2).

#### LD-based tests.

We used the *K* and *ZnS* tests as one-sided tests. We look for a lower-than-expected number of haplotypes (*K* test) or stronger-than-expected association between sites (*ZnS* test). Just like the frequency-spectrum–based tests, the LD tests do not have power to detect a hard sweep at the selected locus at the time of fixation, because there are no polymorphic sites. At some distance from the selected site, both LD tests have high power (up to 69% for *K*) to detect hard sweeps, especially at *R* from 100–600. However, whereas Tajima's *D* performed best for hard sweeps, the LD tests perform better for soft sweeps. Their power increases if the beneficial mutation rate is increased, in particular close to the selected locus. This means, in particular, that recent soft selective sweeps from recurrent mutation, unlike hard sweeps, can be detected from polymorphism data (e.g., from introns) from a selected gene itself. Kelly's *ZnS* test shows roughly the same pattern as the *K* test. *ZnS* is somewhat less powerful at the time of fixation, but its power lasts longer after the sweep. For both *K* and *ZnS,* it should be noted that their power reduces quickly after fixation, and at *t* = 0.1, there is virtually no power left.

#### Effect of further parameters.

We did additional simulation runs for weaker selection (*α* = 1,000) and a different length of the neutral fragment (Θ*_n_* = *R_n_* from 2 to 40). None of these changes affects the qualitative results that we have reported above. For *α* = 1,000, the power of all tests is reduced by several percent, as already reported, e.g., by Przeworski [33]. Also, to compare results, the recombination distance *R* must be rescaled to ≈ *R*/10 to account for the about ten times longer fixation time. Importantly, however, the effect of the beneficial mutation rate and the change in the power of the tests from hard to soft sweeps stays the same.

The power of the frequency-spectrum–based tests generally slightly increases for longer fragments and more strongly decreases for shorter fragments, due to the larger number of polymorphic sites. For tests based on LD (*K* and *ZnS*), there is a clear decrease of power in some cases for fragments with *R_n_* > 10–20. This is expected since recombination within the fragment will reduce LD.

#### Improving the power of the LD-based tests.

The power of the LD-based tests reduces very quickly with time. There are three reasons for this. First, ancestral variation disappears from the population through drift. Since it is ancestral variation that is in LD, tests will only detect significant deviations as long as there is sufficient ancestral variation in the sample. Second, new mutations accumulate, and these mutations are not in LD, nor are they organized in clear haplotypes. Finally, recombination between the ancestral haplotypes can reduce LD and increase the number of haplotypes. In 0.1*N_e_* generations, drift reduces the number of ancestral polymorphic sites by only about 15%, and it seems to be the other two factors that are most important for the reduction of power.

Note that our tests are very conservative in that they assume no recombination for the neutral simulations. If a reliable estimate of the recombination rate is available, neutral simulation with a (conservatively low) *R* > 0 can increase the power significantly [38]. To account for the effect of new mutations, we suggest here a variant of the test that is possible in certain scenarios if data from a sister population are available.

Imagine that we are interested in local adaptations of a colony population to a new “island” habitat and that the “continental” founder population that continues to live under ancestral conditions is also known. Assume further that there is no recent gene flow between the two sister populations. We may then use data from the founder population to identify shared polymorphisms that predate the adaptation. Mutations that are only found in the island population may be new mutations and are taken out of the analysis. Glinka et al. [39], for example, show that 65 of the mutations found in a European D. melanogaster population (the “colony”) are also found in an African sister population, even though the authors have only a small sample from Africa. Under the assumption that there is no gene flow between the populations, we can consider mutations that are found in both populations as ancestral variation.

To see what would be the effect on the power of the different tests of using only ancestral variation, we have done simulations of positive selection in which we have stopped neutral mutational input at the start of the selective phase. For neutral comparison, we stopped mutational input in the last *tN_e_* generations of the tree. The result is promising: the power of the LD tests is much higher if we consider only ancestral variation (see Figure 8). This is even though there are fewer mutations in the analysis (at *t* = 0.1, at the selected site, mean *S* is 16.4 with new mutations and only 9.6 without). The power also increases for hard sweeps in the flanking regions (unpublished data). However, for Tajima's *D,* the method does not work: the power stays low for soft sweeps, and for hard sweeps, the power is higher if we allow for new mutations.

**Figure 8:**
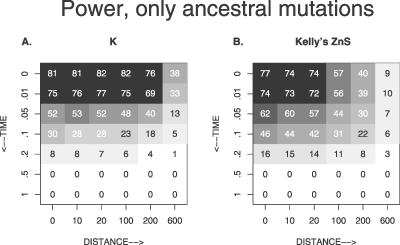
The Percentage of Simulation Runs That Yielded a Significant Test Statistic if We Condition on a Soft Sweep and Ignore Mutations during and after the Sweep θ*_b_* = 0.1, other parameters as standard. The *x-*axis shows the distance from the selected site in units of *R* = *N_e_r*. The *y-*axis shows the time since fixation of the *B* allele in units of *N_e_* generations.

To apply this approach to data, the following steps should be taken. A not-too-divergent sister population is needed and an accurate estimate of the divergence time, *d*. To obtain critical values for the tests, neutral simulations should be done with no mutations in the last *d* generations. The data from the focus population should be compared with a large sample from a large sister population, so that as many mutations as possible can be identified as ancestral. If only a small sample is used, many mutations will have to be taken out of the analysis, making the tests less powerful. Similarly, power is lost if the sister population is small or divergence time too long, such that many variants are lost due to drift.

### Adaptation from Recurrent Mutation

New beneficial alleles can enter a population also by recurrent migration, instead of mutation. In Pennings and Hermisson [24], we have shown that the number and distribution of ancestral haplotypes directly at the selected site (at recombination distance *R* = 0) in this case are again given by the Ewens sampling formula, as in the recurrent mutation case. The mutation rate Θ*_b_* is replaced by the number of migrants per generation *M*. If we assume that the adaptation in the source population is very old, such that migrants are related by a neutral coalescent, the results on the polymorphism pattern at a tightly linked locus, as described above, carry over to the migration scenario (with Θ*_n_* the mutation rate in the source population).

At a linked locus (*R* > 0) near the selected site, haplotypes from both populations may appear in a sample. Depending on the divergence time of the populations, these haplotypes may be much more divergent than haplotypes from a single population. As far as the LD pattern is concerned, the enhanced divergence among haplotypes leads to a clearer footprint of selection. Tests based on LD will therefore have a higher power if adaptation originates from migrants from a divergent source population. Divergence between both populations also has an effect that partly opposes the effect of the sweep. As Santiago and Caballero [40] have shown for a sweep from a single migrational origin, heterozygosity may even be increased above the population average in the flanking regions of the selected site. The same effect will also be visible for a soft selective sweep from recurrent migration.

## Discussion

### Main Results

The main result of our study is that soft sweeps from recurrent mutation leave a clear signature on the neutral DNA polymorphism pattern. For recent sweeps, this pattern may even be clearer than the classical signature of a hard sweep from a single new mutation. This may be surprising because 1) the variation is not as much reduced as in the hard-sweep case (see Figure 5), and 2) the folded frequency spectrum is not much different than the neutral expectation (see Figure 2). In contrast, however, soft sweeps will typically lead to a stronger signal in LD as compared with the classical pattern. This is because a second beneficial mutant brings along with it a complete new haplotype. The presence of two (or more) independent haplotypes causes the polymorphic sites to be in complete LD.

After a recent hard sweep, polymorphism in the direct vicinity of the selected site is often almost completely erased. As a consequence, standard neutrality tests have very little power in this region. Recent positive selection can then only be detected from flanking regions of a selected gene, where ancestral polymorphism is maintained due to recombination. Positive LD, in particular, is also limited to these flanking regions and usually does not extend across the selected locus [7,41]. In contrast, for a soft sweep from recurrent mutation, polymorphism in the shape of several ancestral haplotypes is maintained directly at the selected locus. This leads to strongly positive LD which extends to both sides of the selection center. Tests based on LD therefore have a high power over long stretches of DNA, including the selection locus. Because genes are a common selection target, and most available data are from genes, we expect that soft sweeps may indeed be easier to detect than hard sweeps.

For the classical signature of a hard sweep, Kim and Stephan [11] and Kim and Nielsen [41] have shown that most information is contained in the frequency spectrum. Adding LD to the analysis does not increase the power of a neutrality test much further [41]. We find that essentially the opposite is true for the pattern of a soft selective sweep from recurrent mutation. Soft sweeps are characterized by the LD pattern and not by the frequency spectrum. For the classical test based on the frequency spectrum, Tajima's *D,* we find that the mean hardly deviates from neutrality, and the variance is much increased relative to both neutrality and the classical hard sweep. The reason for the conspicuous difference to a hard sweep, in which recombination leads to a negative *D,* lies in the timing of these events during the selective phase. Although recombination typically happens later than coalescence (in a forward-in-time picture), and therefore produces low-frequency variants in a sample, recurrent beneficial mutation happens at the same time as coalescence. It can therefore either affect single branches (leading to a negative Tajima's *D*) or larger families of branches that have already coalesced (leading to positive Tajima's *D* values). The variance in *D* that results is even higher than for the case of a selective sweep from the standing genetic variation, in which a similar phenomenon has been observed [21,22]. Indeed, as our Figure 7 shows, we expect a significantly negative or positive Tajima's *D,* each in 20% of cases, for a recent soft sweep and data from the selected locus. Importantly, this demonstrates that significantly positive *D* is not incompatible with positive selection under this scenario.

The inverse roles of the frequency spectrum and the LD pattern for hard and soft selective sweeps suggest a dual approach to detect positive selection in genome scans. A standard frequency-based test, such as Tajima's *D,* should be combined with a LD test like *ZnS* (given that the phase information is available), in particular if the effective population size and allelic mutation rates are likely to be large or if adaptation from recurrent migration could play a role. We note that an untypical signature of positive selection with strong positive LD across the selected site (as in the case of a soft sweep) could also result from hard sweep if there is gene conversion (see also [13]). For this, we need to assume that gene conversion happens during the selective phase and that the gene conversion tract includes the selected site.

Although high levels of LD are a strong signal of a recent soft sweep from recurrent mutation, the pattern quickly fades for older sweeps, due to new mutations and recombination (see Figure 7). Here, we find that the power of LD-based tests is greatly increased if new mutations can be taken out of the analysis. This is possible if polymorphism data from the same locus from a recently diverged sister population are available. One can then include only shared polymorphisms into the analysis, which effectively purges the study population of all mutations that occurred after the split. For practical use, the divergence time between the populations needs to be estimated, and critical values for the test statistics need to be obtained from neutral simulations with no mutations since the divergence of the populations. The method works best if the sister population is large, if a large sample is available from the sister population, and if the divergence between the populations has occurred not too long before the start of positive selection in the study population. In this case, we obtain a high power of neutrality tests based on LD for about 0.1*N_e_* generations, which is comparable to the values for Tajima's test for the classical sweep pattern (see Figure 8).

### Conditions and Caveats

Throughout this study, we have assumed that the population in which we want to detect selection is panmictic with a constant size. It is well-known that population structure and demography can mimic the polymorphism patterns that are typical of positive selection. This is true for the classical sweep pattern, in which population growth or bottlenecks are alternative mechanisms that can produce an excess of rare alleles. It also holds for the signature of a soft sweep from recurrent mutation. Strong positive LD can result, for example, from bottlenecks and from admixture [42,43]. Ignoring population demography can therefore lead to a high rate of false positives in the neutrality tests. The general strategy to overcome this problem, at least partly, is to compare data from candidate loci with genome-wide data to account for demographic effects (cf. [12,17,19,44]). Another scenario that is known to produce significantly positive LD is balancing selection. However, long-term balancing selection would lead to a haplotype structure in which each of the haplotypes carries neutral variation. In contrast, the haplotypes after a soft sweep should contain only very little variation from new mutations, which should make it possible to distinguish these two scenarios.

One important assumption of our model is that the beneficial allele can only arise at a single locus. In some cases, this may not be the case. For example, several mutations at different loci may affect the efficiency of a pathway in the same way. In the ancestral genetic background, all these mutations then have an equivalent effect on phenotype and fitness. In the presence of one of these mutations, a second mutation at a different locus may be neutral. If two of these mutations at different loci are picked up by selection and simultaneously increase in frequency, they will at some point start to interfere with each other. Fixation of the allele at one locus will stop the frequency increase at the other locus, leading to the pattern of a partial sweep.

We have also assumed that all variants of the beneficial allele have exactly the same fitness effect, which may be unrealistic. However, in Pennings and Hermisson [24], we have looked at the effect of variable selection coefficients across the distribution of ancestral haplotypes and found that the effect is limited as long as this variation is not very strong. We therefore expect that the results in this paper will also remain robust under moderate differences in selection coefficient (*s*). Similarly, we expect that all results that depend on the distribution of ancestral haplotypes due to recurrent mutation are robust to relaxations of various other model assumptions, which are all discussed in Pennings and Hermisson [24]. In particular, this holds for diploidy, frequency-dependent selection or dominance, changing selection pressures, and for adaptation from standing genetic variation.

### Data

Patterns of soft selective sweeps from recurrent mutation have not been the focus in genome scans for positive selection so far. Nevertheless, there are several examples in published data that are suggestive of soft sweeps. The clearest case comes from three immunity receptor genes in D. simulans and was reported by Schlenke and Begun [19]. All three genes show extreme levels of LD due to two major haplotypes that have not recombined. In one case, there is a third haplotype at low frequency. Although there are normal levels of variation among haplotypes, there is no variation within the haplotype classes, with the exception of a single singleton in one case. In accordance with our expectations for soft sweeps from recurrent mutation, frequency-spectrum–based tests did not result in significant values. However, when the authors used the *ZnS* test, all three genes were highly significant and were clear outliers relative to reference samples from other genes. The authors found that a bottleneck could not explain the high *ZnS* values. Since LD is maximal on the gene, but quickly decreases both upstream and downstream, the authors conclude that the gene itself has been the target of positive selection. As mentioned above, gene conversion during a hard sweep offers an alternative explanation for strongly positive LD that extends to both sides of a selection center. This seems possible in one of the genes *(Tehao),* where in the middle of the gene there is a stretch of 1,300 bp without any polymorphism. However, no such stretch without polymorphism is visible for the other two genes. Together with the absence of a signal in the frequency spectrum, this makes soft sweeps from recurrent origins the most plausible explanation.

A second example is the Duffy locus in humans. The *FY-0* allele at this locus confers resistance against malaria and is found at near fixation in sub-Saharan African populations, but is very rare everywhere else [13]). Also, the responsible mutation is known. This mutation is found on two different haplotypes, which are characterized by a single nucleotide polymorphism (SNP) and an indel on the 5′ side of the beneficial mutation and a SNP on the 3′ side. Because the haplotypes are characterized by few SNPs, and because there are some singletons in the region as well, no test statistic is significant for this locus. However, other data, such as a very high *F_ST_* value, strongly support the hypothesis that the *FY-0* allele rose to fixation because of selection. This, combined with the two haplotypes that are seen, makes a soft sweep a plausible explanation, although a hard sweep with a gene conversion is an alternative scenario. In Hamblin et al. [45], evidence was found for a hard sweep associated with the *FY-0* allele in the Hausa population. However, this population was chosen for this study because it had only one of the two haplotypes.

As an illustration of the method that we suggest, we present data from a fragment on the X chromosome from a European and an African sample of D. melanogaster (Figure 9). This fragment (fragment 163 from [17]) has nine polymorphic sites in the European fragment, and neither frequency-spectrum–based tests nor LD tests show a deviation from neutrality. However, the six polymorphisms that are shared between Europe and Africa are in perfect LD in the European sample. When only considering the shared polymorphisms, there are two perfect haplotypes of which one is found five times and one is found seven times in the sample. Both the *ZnS* test and the *K* test show significant deviation from neutrality.

**Figure 9:**
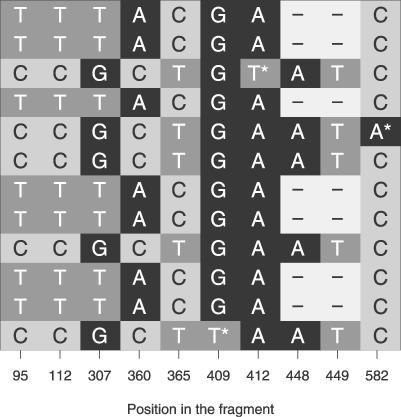
Polymorphic Sites in a Fragment on the X Chromosome of a Sample from Drosophila melanogaster in a Sample from Europe The polymorphic sites that are unique to the European sample are indicated by an asterisk (*). The indel of 2 bp is counted as one polymorphic site.

LD or haplotype structure is used by many studies to find alleles that have recently increased in frequency. As long as the allele has not reached fixation, the region around the locus will show strong LD [7]. Sabeti et al. [46] developed a method to use this pattern of strong LD to identify local or partial sweeps. A modified version of the Sabeti method was applied to HapMap data by Voight et al. [47] to identify partial sweeps. Complete hard sweeps cannot be detected by this method, but with a slightly altered version of this method, it should be possible to use HapMap data to detect soft sweeps.

### Analytical Derivations

#### Frequency distribution of ancestral variation.

In this section, we derive the frequency distribution of ancestral neutral polymorphisms at a tightly linked neutral locus after a soft selective sweep from recurrent mutation. This means we assume that no recombination during the selective phase has happened between the selected site and the locus studied. We focus on the contribution of ancestral variation to the frequency spectrum and thus ignore new mutations (neutral mutations that have occurred after the start of the selective phase).

Assume that we take a sample of size *n* directly (or sufficiently soon) after fixation of a beneficial allele that enters the population with a mutation parameter Θ*_b_* = 2*uN_e_*. In Pennings and Hermisson [24], we have shown that the distribution of ancestral haplotypes in such a sample follows the Ewens sampling formula. For the frequency spectrum of ancestral polymorphisms, we need to combine this result with a neutral coalescence process of the surviving ancestral haplotypes for the time prior to the selective phase. We need the following ingredients for a derivation:

First, according to the Ewens sampling formula, the probability for *k* ancestral haplotypes in the sample is

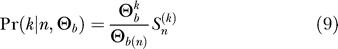
where we define 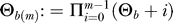 , and 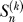 is the nonnegative Stirling number of first kind

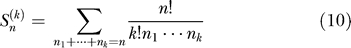
which counts the number of permutations of *n* objects with *k* permutation cycles 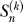 = 1; 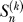 = 0 for *k* > *n*). Since there are no ancestral polymorphisms if there is only a single ancestral haplotype, *k* = 1, we need to condition on *k* > 1,

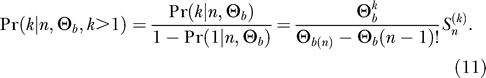
Second, the probability that the derived variant appears in *j* out of *k* haplotypes is

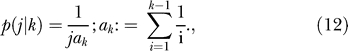
given that the population is in neutral equilibrium. If this is not the case, an empirical frequency spectrum estimated from genome-wide data can be used instead (as in [12]). And third, again according to the Ewens sampling formula, the probability for a haplotype distribution of {*n*_1_,...,*n_k_*}, given that *k* haplotypes are found in a sample of size *n* is

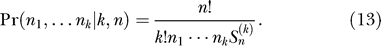
Assume now that *j* out of *k* haplotypes carry the derived mutation. The probability that ℓ individuals out of *n* carry the derived mutation under this condition then gets

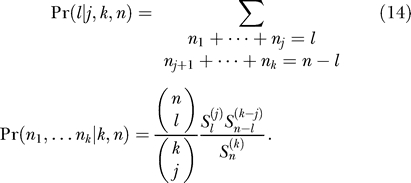
We can now combine all these components to obtain the ancestral polymorphism spectrum as

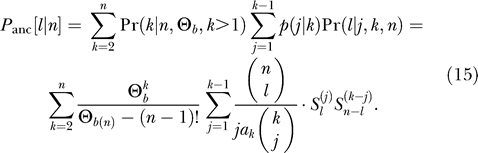
where ℓ + *k* − *n* ≤ *j* ≤ ℓ. Conditioned on a soft sweep with *k* haplotypes we obtain:

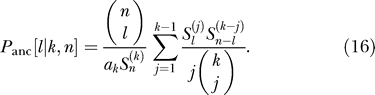
An interesting consequence is that the ratio of singletons to (*n* − 1) letons is (*k* − 1) to 1. So, if *k* = 2, the frequency spectrum is symmetrical.

#### Distribution of distinct ancestral haplotypes.

Ancestral haplotypes are not necessarily distinct since they might be identical by descent. For the probability to obtain *l* distinct ancestral haplotypes, given that there are *k* ancestral haplotypes, we need to follow these haplotypes in a neutral coalescent process with mutations prior to the selective phase. The number (and distribution) of distinct haplotypes is then again given by the Ewens sampling formula, this time on a sample of size *k* and with the neutral mutation rate Θ*_n_* on the fragment, i.e., by 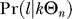 using Equation 9. For the entire probability to obtain *l* distinct ancestral haplotypes, we thus need to combine two Ewens sampling steps to obtain

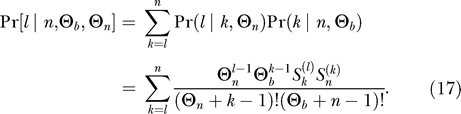



####  The expected number of polymorphic sites.

We assume that lineages escape independently by recombination. Using Equation 7, we thus obtain the probability that *q* lineages escape through recombination as a binomial

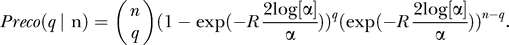



 The probability that there are *k* ancestors for the *n − q* lineages that have not escaped through recombination is given by 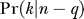 (Equation 9). The probability that there are *m* independent haplotypes in total is therefore given by

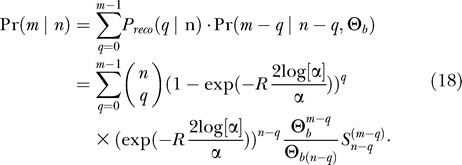



## Abbreviations

LD: linkage disequilibrium
